# Rod-like anhydrous V_2_O_5_ assembled by tiny nanosheets as a high-performance cathode material for aqueous zinc-ion batteries†

**DOI:** 10.1039/c9ra06143f

**Published:** 2019-09-26

**Authors:** Weijun Zhou, Jizhang Chen, Minfeng Chen, Xinwu Xu, Qinghua Tian, Junling Xu, Ching-Ping Wong

**Affiliations:** College of Materials Science and Engineering, Nanjing Forestry University Nanjing 210037 China jizhang.chen@hotmail.com; Department of Chemistry, School of Sciences, Zhejiang Sci-Tech University Hangzhou 310018 China; Department of Electronic Engineering, The Chinese University of Hong Kong NT Hong Kong China junlingxu@outlook.com; School of Materials Science and Engineering, Georgia Institute of Technology Atlanta USA

## Abstract

Aqueous zinc-ion batteries offer a low-cost and high-safety alternative for next-generation electrochemical energy storage, whereas suitable cathode materials remain to be explored. Herein, rod-like anhydrous V_2_O_5_ derived from a vanadium-based metal–organic framework is investigated. Interestingly, this material is assembled by tiny nanosheets with a large surface area of 218 m^2^ g^−1^ and high pore volume of 0.96 cm^3^ g^−1^. Benefiting from morphological and structural merits, this material exhibits excellent performances, such as high reversible capacity (449.8 mA h g^−1^ at 0.1 A g^−1^), good rate capability (314.3 mA h g^−1^ at 2 A g^−1^), and great long-term cyclability (86.8% capacity retention after 2000 cycles at 2 A g^−1^), which are significantly superior to the control sample. Such great performances are found to derive from high Zn^2+^ ion diffusion coefficient, large contribution of intercalation pseudocapacitance, and fast electrochemical kinetics. The *ex situ* measurements unveil that the intercalation of Zn^2+^ ion is accompanied by the reversible V^5+^ reduction and H_2_O incorporation. This work discloses a direction for designing and fabricating high-performance cathode materials for zinc-ion batteries and other advanced energy storage systems.

## Abstract 2

V_2_O_5_ with intriguing micro/nano-hierarchical structure is fabricated *via* the pyrolysis of MIL-47 (a MOF material) and displays great performances as the cathode material for aqueous zinc-ion batteries.

## Graphical abstract

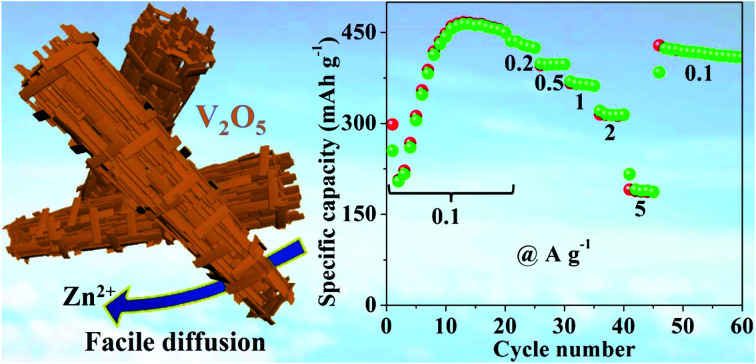

## 1. Introduction

Although lithium-ion batteries (LIBs) have dominated in portable electronics and emerging electric vehicles, their future potential for large-scale energy storage systems is strongly hindered by limited lithium resources, high cost, and safety issues.^1–3^ With the advantages of low cost, good safety, and high energy density, aqueous zinc-ion batteries (AZIBs) are becoming an alternative technology to LIBs, especially for large-scale applications.^3–6^ On the one hand, zinc element is abundant on the earth, and its metallic form is stable in water and has an approximate redox potential of −0.76 V *vs.* standard hydrogen electrode (SHE), allowing metallic Zn to be directly used as the anode of AZIBs with a large theoretical specific capacity (820 mA h g^−1^ and 5855 mA h cm^−3^).^4,7^ On the other hand, aqueous electrolytes contribute to better safety, higher ionic conductivity, easier processing, and lower cost in comparison with organic electrolytes.^5^ Despite great advances made for AZIBs in the last several years, AZIBs are still in their infancy, and their development is severely restricted by the unsatisfactory cathode materials, mainly due to heavy mass and high polarization of divalent Zn^2+^ ion.^1,3^

In recent years, Mn-based oxides (*e.g.*, MnO_2_,^8–12^ Mn_3_O_4_,^13^ and ZnMn_2_O_4_ (ref. 14)), polyanionic compounds (*e.g.*, Na_3_V_2_(PO_4_)_2_F_3_ (ref. 15) and LiV_2_(PO_4_)_3_ (ref. 16)), Prussian blue analogues,^17^ Mo-based compounds (*e.g.*, MoS_2_ (ref. 18)), and organic and polymer compounds (*e.g.*, polyaniline^19–21^ and *p*-chloranil^22^) have been investigated as the cathode materials for AZIBs. However, these materials suffer from either low specific capacity or poor rate capability or short life span. Very recently, vanadium oxide, sulfide, and vanadate cathode materials have attracted much attention since Nazar *et al.* reported Zn_0.25_V_2_O_5_·*n*H_2_O as a high-capacity (∼300 mA h g^−1^) and long-life (1000 cycles) cathode material.^23–31^ It is noteworthy that the reversible, stable, and rapid redox reactions associated with vanadium element render these materials highly appealing for AZIBs. For example, Alshareef *et al.* developed hydrated layered Mg^2+^-intercalated V_2_O_5_ (Mg_0.34_V_2_O_5_·0.84H_2_O) with a large interlayer spacing of 13.4 Å, which enables high capacities of 353 and 264 mA h g^−1^ at 0.1 and 1 A g^−1^, respectively.^25^ Niu *et al.* reported NaV_3_O_8_·1.5H_2_O nanobelts, which exhibit high capacity of 380 mA h g^−1^ at 0.05 A g^−1^ and great capacity retention of 82% over 1000 cycles.^30^

Among various vanadium-based materials, anhydrous V_2_O_5_ is of particular interest owing to its simple configuration and high valence state of vanadium that enable more active sites and higher specific capacity.^32–40^ That is, anhydrous V_2_O_5_ is free of cations (*e.g.*, Zn^2+^, Li^+^, Na^+^, K^+^, Mg^2+^, Ca^2+^) and H_2_O molecules within its layers, thus possessing higher gravimetric and volumetric specific capacities than other vanadium-based materials in theory, while such characteristic also makes Zn^2+^ ion uptake difficult due to the limited interlayer space.^34,41,42^ For instance, porous V_2_O_5_ nanofibers prepared by electrospinning and subsequent calcination delivers merely 319 mA h g^−1^ at a rather low current density of 0.02 A g^−1^, which is substantially lower than the theoretical specific capacity (∼589 mA h g^−1^ based on the two-electron redox center of vanadium) of V_2_O_5_.^34^ In addition, this V_2_O_5_ shows low capacities at high current densities, *e.g.*, 104 mA h g^−1^ at 2.94 A g^−1^.^34^ As is widely utilized for electrode materials, tailoring the morphology, size, and porosity is an effective way to address the above-mentioned problem. In the present work, in order to demonstrate the importance of morphology engineering for V_2_O_5_ cathode material and elucidate its Zn^2+^ ion uptake mechanism, we focus on rod-like anhydrous V_2_O_5_ (denoted RA-V_2_O_5_) that is fabricated *via* the pyrolysis of MIL-47. MIL-47 is a vanadium-based metal–organic framework (MOF) V^IV^(O)(bdc), in which bdc represents 1,4-benzenedicarboxylate. It is found that RA-V_2_O_5_ possesses micro/nano-hierarchical structure with large specific surface area and high pore volume, consequently displaying great electrochemical performances.

## 2. Experimental

### 2.1. Materials synthesis and characterization

MIL-47 was synthesized according to a previous report.^43^ The as-obtained MIL-47 was kept in a furnace at 350 °C for 4 h in the air atmosphere, thus producing RA-V_2_O_5_. The morphology and microstructure were characterized on JEOL JSM-7600F field emission scanning electron microscope (FE-SEM), JEOL JEM-2100UHR transmission electron microscope (TEM), and Quantachrome Autosorb-iQ2-MP N_2_ adsorption/desorption analyzer. The crystallographic information, phase purity, and chemical compositions of the samples were collected by Rigaku Ultima IV powder X-ray diffractometer (XRD) with Cu Kα radiation source, Elementar Vario EL Cube elemental analyzer, TA Instruments Q5000 IR thermogravimetric analyzer (TGA), Thermo Scientific DXR Raman Spectrometer with *λ* = 532 nm laser excitation, and Kratos AXIS UltraDLD X-ray photoelectron spectrometer (XPS).

### 2.2. Electrochemical measurements

RA-V_2_O_5_, Super-P carbon black, and polyvinylidene difluoride (PVDF) were mixed in *N*-methylpyrrolidone (NMP) homogeneously at a weight ratio of 7 : 2 : 1 to form a slurry, which was casted on a titanium foil and dried at 80 °C for 4 h. Then the titanium foil was pressed at 10 MPa and cut into round pieces (12 mm in diameter) as working electrodes. The mass loading of RA-V_2_O_5_ is 1.5–2 mg cm^−2^. CR2016 coin cells were assembled by a traditional method using glass fiber membrane (Whatman GF/A), zinc foil (0.25 mm), and 3 M Zn(CF_3_SO_3_)_2_ aqueous solution as the separator, counter electrode, and electrolyte, respectively. The electrochemical performances of coin cells were examined at current densities from 0.1 to 5 A g^−1^ in the potential window from 0.2 to 1.6 V *vs.* Zn^2+^/Zn using a LAND CT2001A battery test system. Cyclic voltammetry (CV) and electrochemical impedance spectroscopy (EIS) measurements were carried out using a CHI 660E electrochemical workstation. The solubility of RA-V_2_O_5_ in the electrolyte was evaluated by inductively coupled plasma mass spectrometer (ICP-MS).

## 3. Results and discussion

**Fig. 1:**
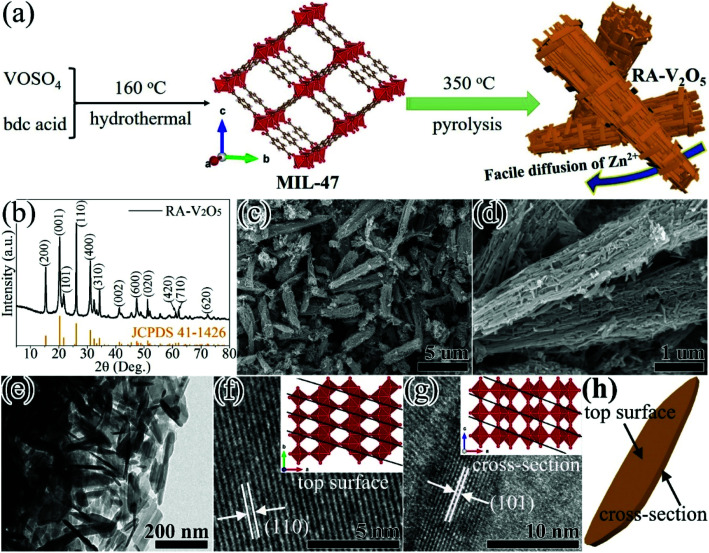
(a) Schematic illustration of the fabrication process, (b) XRD pattern, (c and d) SEM images, (e–g) TEM images, and (h) illustration of the nanosheet top surface and cross-section of the RA-V_2_O_5_.

RA-V_2_O_5_ was fabricated through a two-step process (see Fig. 1a). In the first step, MIL-47 was prepared by a facile hydrothermal reaction of VOSO_4_ and bdc acid at 160 °C.^43^ In the second step, the as-obtained MIL-47 was pyrolyzed at 350 °C for 4 h in air, thus transforming into RA-V_2_O_5_. The crystalline phase of RA-V_2_O_5_ was verified by XRD, as shown in Fig. 1b, which matches exactly with the orthorhombic shcherbinaite V_2_O_5_ with a *Pmmn* space group (JCPDS 41-1426). The intense peaks at 2*θ* = 15.35°, 20.25°, 21.68°, 26.11°, 31.00°, and 34.30° correspond to the (200), (001), (101), (110), (400), and (310) planes of V_2_O_5_, respectively. According to the elemental analysis result, the weight percentages of C and H elements are both lower than 0.3% in RA-V_2_O_5_, indicating RA-V_2_O_5_ is almost free of carbon and water. The anhydrous nature of RA-V_2_O_5_ is also confirmed by the TGA curve in Fig. S1.† For comparison, commercial V_2_O_5_ purchased from Aladdin (denoted C-V_2_O_5_) was also investigated as the cathode material for AZIBs. As can be observed from Fig. S2,† all the XRD peaks of C-V_2_O_5_ are attributed to shcherbinaite V_2_O_5_. It is also seen that the peaks of C-V_2_O_5_ are much sharper than that of RA-V_2_O_5_, indicative of much lower crystallinity of the latter.

Fig. 1c shows the global SEM image of RA-V_2_O_5_, revealing a homogeneous rod-like morphology. These rods are around 1 μm in diameter and have extremely rough surface. It can be clearly seen from Fig. 1d and e that RA-V_2_O_5_ rods consist of interconnected tiny nanosheets with the thickness of 5–15 nm. Notably, there exist sufficient voids among RA-V_2_O_5_ nanosheets, which can considerably facilitate Zn^2+^ ion diffusion within V_2_O_5_, therefore realizing high specific capacity and great rate capability. Besides, these voids can effectively accommodate the volumetric expansion of V_2_O_5_ upon Zn^2+^ ion uptake, which is beneficial for cycling stability. The high-resolution (HR) TEM images of RA-V_2_O_5_ nanosheet at the top surface and cross-section are presented in Fig. 1f and g, in which the lattice fringes of 0.337 and 0.406 nm correspond to the (110) and (101) crystalline planes of shcherbinaite V_2_O_5_, respectively. The orientations and locations of these two planes are illustrated in Fig. 1h and the inset of Fig. 1f and g. As for C-V_2_O_5_, it is comprised by large particles of 200–600 nm in size, as shown in Fig. S3.†

**Fig. 2:**
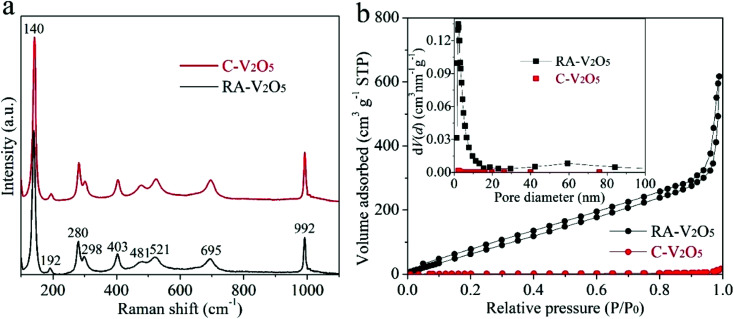
(a) Raman spectra and (b) N_2_ adsorption/desorption isotherms of RA-V_2_O_5_ and C-V_2_O_5_ (the inset of (b) shows corresponding BJH pore-size distributions).

Fig. 2a displays Raman spectra of two V_2_O_5_ samples. They show nearly identical profiles, and are in good consistent with previously reported V_2_O_5_.^33,34^ Specifically, the nine bands can be divided into three regions, *i.e.*, 500–1000 cm^−1^, 200–500 cm^−1^, and the strongest band at 140 cm^−1^, originating from the bond stretching modes, the angle bending modes, and the shear motion and rotations of the ladders along their axes, respectively.^44^ The high purity of RA-V_2_O_5_ is also confirmed by its XPS spectrum (Fig. S4†), which only contains peaks belonging to V and O. As for C-V_2_O_5_, a small amount of Na is detected. The existence of sufficient voids within RA-V_2_O_5_ is evidenced by N_2_ adsorption–desorption analysis. As is shown in Fig. 2b, RA-V_2_O_5_ exhibits type IV isotherm (IUPAC classification), indicative of mesoporous structure. According to Brunauer–Emmett–Teller (BET) theory, the specific surface area of RA-V_2_O_5_ is measured to be 218 m^2^ g^−1^, much higher than that of C-V_2_O_5_ (5.3 m^2^ g^−1^). It is worthwhile mentioning that the specific surface area of RA-V_2_O_5_ is significantly larger than that of previously reported V_2_O_5_ cathode materials for AZIBs, *e.g.*, porous V_2_O_5_ nanofibers (27.1 m^2^ g^−1^),^34^ V_2_O_5_ hollow spheres (11.1 m^2^ g^−1^),^37^ and V_2_O_5_ nanofibers (17.9 m^2^ g^−1^),^38^ therefore making electrolyte permeation and Zn^2+^ ion insertion more favourable within RA-V_2_O_5_. Besides, a H_3_-type hysteresis loop is observed for RA-V_2_O_5_, suggesting it is composed of sheet-shaped particles, which accords well with its actual morphology observed from TEM. The mesoporous nature of RA-V_2_O_5_ is further supported by the Barrett–Joyner–Halenda (BJH) pore size distribution, as shown in the inset of Fig. 2b. In particular, a well resolved peak centered at *ca.* 2.3 nm can be observed. And RA-V_2_O_5_ has a large pore volume of 0.96 cm^3^ g^−1^, in comparison with merely 0.01 cm^3^ g^−1^ of C-V_2_O_5_.

**Fig. 3:**
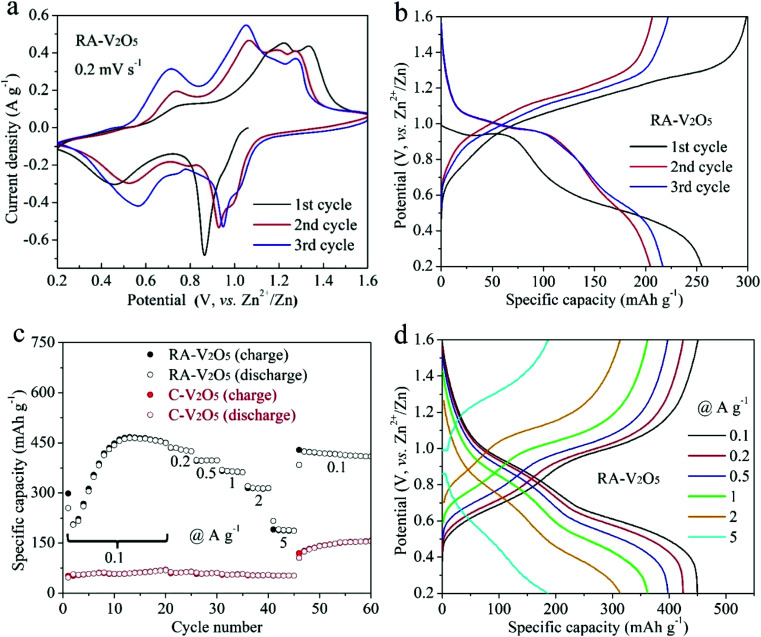
(a) CV curves of RA-V_2_O_5_ in initial three cycles at 0.2 mV s^−1^. (b) GCD curves of RA-V_2_O_5_ in initial three cycles at 0.1 A g^−1^. (c) Rate performances of RA-V_2_O_5_ and C-V_2_O_5_ at current densities from 0.1 to 5 A g^−1^. (d) GCD curves of RA-V_2_O_5_ at different current densities.

The electrochemical properties of RA-V_2_O_5_ and C-V_2_O_5_ were evaluated in a coin-cell configuration using zinc metal foil and 3 M Zn(CF_3_SO_3_)_2_ aqueous solution as the counter/reference electrode and electrolyte, respectively. The CV curves of RA-V_2_O_5_ in the initial three cycles at a scan rate of 0.2 mV s^−1^ in the potential window of 0.2 to 1.6 V (*vs.* Zn^2+^/Zn) are shown in Fig. 3a. Multiple pairs of redox peaks can be observed, demonstrating a multistep Zn^2+^ ion uptake/release process in V_2_O_5_ cathode materials for AZIBs.^36–39^ The CV curves of RA-V_2_O_5_ at the 1^st^ cycle is different from that in the following cycles in terms of peak positions and profiles, behind which the possible reason is the activation of V_2_O_5_. It is also seen that RA-V_2_O_5_ exhibits more peaks than C-V_2_O_5_ (Fig. S5†) and the peak current densities of RA-V_2_O_5_ are much stronger, unveiling that RA-V_2_O_5_ has higher electrochemical reactivity than C-V_2_O_5_. Fig. 3b presents the galvanostatic charge/discharge (GCD) curves of RA-V_2_O_5_ in initial three cycles at the current density of 0.1 A g^−1^, in which sloped plateaus can be observed. And the characteristics of these curves are consistent with the above-mentioned CV curves. As for C-V_2_O_5_, its GCD curves (see Fig. S6†) shows similar characteristics, whereas its capacities are much lower than that of RA-V_2_O_5_.

Rate capability is an important parameter for practical applications. Fig. 3c exhibits charge and discharge capacities of RA-V_2_O_5_ and C-V_2_O_5_ at current densities from 0.1 to 5 A g^−1^. At 0.1 A g^−1^, the discharge capacity of RA-V_2_O_5_ rises from 2^nd^ cycle gradually, and reaches a maximum value of 464.4 mA h g^−1^ at the 13^th^ cycle. Such phenomenon is ascribed to the electrochemical activation of V_2_O_5_ during Zn^2+^ ion storage, which was also observed for previously reported V_2_O_5_ cathode materials.^32,34,37,40^ The activation is probably related to the poor electrical conductivity of V_2_O_5_,^45^ as well as phase transformation and morphology evolution of V_2_O_5_ during repeated cycles. This process enables more actives sites that were previously inaccessible to Zn^2+^ ions. Then, as the finite activation process becomes slight, the capacity slowly drops to 449.8 mA h g^−1^ at the 20^th^ cycle. As the current density is raised progressively to 0.2, 0.5, 1, 2, and 5 A g^−1^, the discharge capacity of RA-V_2_O_5_ declines to 424.5, 397.8, 361.9, 314.3, and 186.8 mA h g^−1^ at the end of each current density, respectively. When the current density recovers to 0.1 A g^−1^, RA-V_2_O_5_ is able to give high capacities between 410 and 420 mA h g^−1^, indicative of good electrochemical stability. Besides, it is noted that RA-V_2_O_5_ delivers considerably higher capacities than C-V_2_O_5_ under all the current densities, manifesting our construction of RA-V_2_O_5_ is highly meaningful. Remarkably, thanks to the morphological and structural merits, the RA-V_2_O_5_ outperforms many vanadium-based cathode materials reported to date for AZIBs, such as the ones listed in Table S1.† It is clearly seen that our RA-V_2_O_5_ is significantly superior to porous V_2_O_5_ nanofibers (104 mA h g^−1^ at 3 A g^−1^),^34^ V_2_O_5_ nanosheets (100 mA h g^−1^ at 2 A g^−1^),^36^ V_2_O_5_ nanospheres (138.3 mA h g^−1^ at 5 A g^−1^),^39^ and V_2_O_5_ hollow spheres (147 mA h g^−1^ at 5 A g^−1^).^37^ As is depicted in Fig. 3d, the GCD curve profiles of RA-V_2_O_5_ at various current densities display nearly the same shape, implying small polarization and fast electrochemical kinetics.

**Fig. 4:**
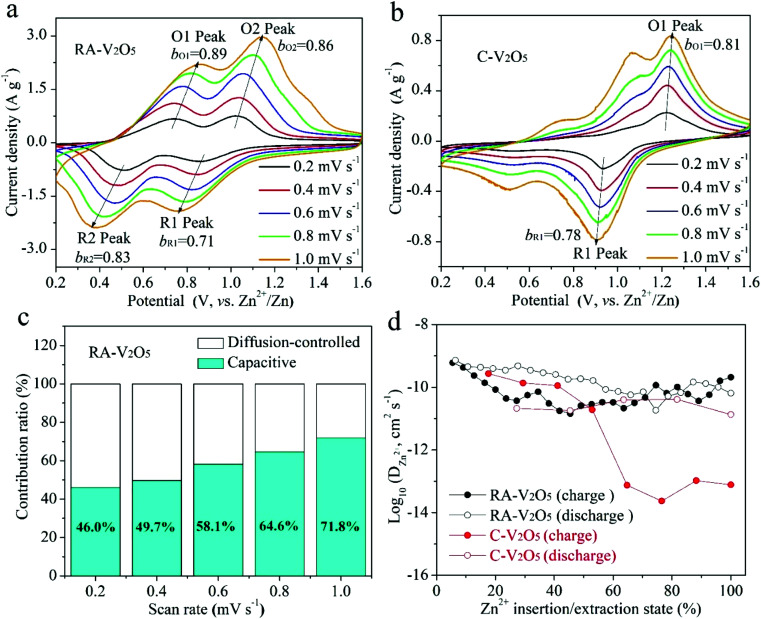
CV curves of (a) RA-V_2_O_5_ and (b) C-V_2_O_5_ at various scan rates. (c) Contribution ratios of diffusion-controlled and capacitive capacities of RA-V_2_O_5_ at different scan rates. (d) *D*_Zn^2+^_ values of RA-V_2_O_5_ and C-V_2_O_5_ determined from the GITT method.

CV measurements were conducted at various scan rates to study the electrochemical kinetics of RA-V_2_O_5_ and C-V_2_O_5_, as displayed in Fig. 4a and b. With the progressive increase of the scan rate from 0.2 to 1.0 mV s^−1^, the anodic peaks slightly move to higher potential, while the cathodic peaks shift to the opposite direction. In the meantime, these peaks become broader gradually. This phenomenon is common for electrode materials, and results from aggravated electrochemical polarization at higher scan rates. When the scan rate is high, the transport of electrons and the diffusion of electrolyte ions in the batteries cannot be synchronized with the quick electron transfer in the external circuit. As a result, the potential/voltage during charging would rise, while the potential/voltage during discharge would decrease. If assuming that the relationship between the scan rate (*ν*) and peak current density (*i*) conforms to the following classic power-law equation,^46^ the charge storage kinetics can be estimated:

1 *i* = *aν*^*b*^

where the *b* value can be determined from log(*i*) *vs.* log(*ν*) plots (see Fig. S7†) and has been widely used to distinguish different charge storage mechanisms. A *b* value of 0.5 illustrates the Zn^2+^ uptake/release is controlled by the semi-infinite diffusion of Zn^2+^ ion, while *b* = 1.0 indicates surface-induced capacitive charge storage. As can be seen from Fig. 4a, the *b* values of the O1, O2, R1, and R2 peaks are fitted to be 0.89, 0.86, 0.71, and 0.83, respectively, suggesting the Zn^2+^ ion uptake/release is dominated by capacitive behavior, namely pseudocapacitive intercalation proposed by Dunn *et al.*^46^ Specifically, the *b* values of RA-V_2_O_5_ are much larger than that of previously reported V_2_O_5_,^37,38^ meaning that the Zn^2+^ ion uptake/release is more favoured in our product. In comparison, the *b* values associated with the O1 and O2 peaks of C-V_2_O_5_ are calculated to be 0.81 and 0.78, respectively (see Fig. 4b), which are slightly lower than that of RA-V_2_O_5_. However, the peak intensities of C-V_2_O_5_ are much lower. In addition, the fraction of the response current density *i*(*V*) that is related to pseudocapacitive intercalation could be calculated according to the following equation:^25,29,47,48^

2 *i*(*V*) = *k*_1_*ν* + *k*_2_*ν*^1/2^

where *k*_1_*ν* and *k*_2_*ν*^1/2^ belong to capacitive and diffusion-controlled signals, respectively. The calculation results for RA-V_2_O_5_ are displayed in Fig. 4c. It is observed that the contribution ratio of pseudocapacitive intercalation increases from 46.0% to 71.8% when the scan rate rises from 0.2 to 1.0 mV s^−1^, confirming that the capacitor-like pseudocapacitive intercalation dominates the charge storage process of RA-V_2_O_5_ at high rates. This result elucidates why RA-V_2_O_5_ can give great rate performances.

The Galvanostatic Intermittent Titration Technique (GITT) was used to estimate the Zn^2+^ ion diffusion coefficients (*D*_Zn^2+^_) in RA-V_2_O_5_ and C-V_2_O_5_.^49^ Before GITT tests, the coin cells were run at 0.1 A g^−1^ for 20 cycles to obtain a stable state. Subsequently, a galvanostatic pulse of 1200 s (0.05 A g^−1^) followed by a relaxation of 180 min to allow the potential to reach the equilibrium was repeatedly applied during the whole charge/discharge process. On the basis of the following equation, the *D*_Zn^2+^_ could be calculated:^23,49^3
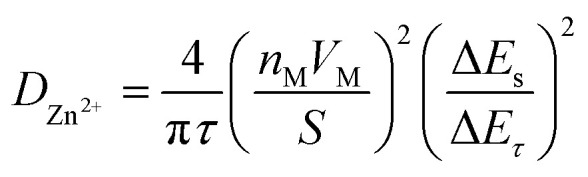


Although the divalent nature of Zn^2+^ ion has an adverse effect on its diffusion within the electrode material, the *D*_Zn^2+^_ of RA-V_2_O_5_ is not low, ranging from 10^−9^ to 10^−11^ cm^2^ s^−1^ (Fig. 4d), which is much higher than Li^+^ ion diffusion in LiFePO_4_ (ref. 50) and Li_4_Ti_5_O_12_,^51^ and higher than *D*_Zn^2+^_ of previously reported V_2_O_5_.^38^ Besides, the *D*_Zn^2+^_ of RA-V_2_O_5_ is slightly higher than that of C-V_2_O_5_ in general, due to much larger specific surface area and pore volume of the former. That is, the ion diffusion at the surface and interface is much more favourable than that within the bulk, therefore contributing to improved rate performances in RA-V_2_O_5_.

**Fig. 5:**
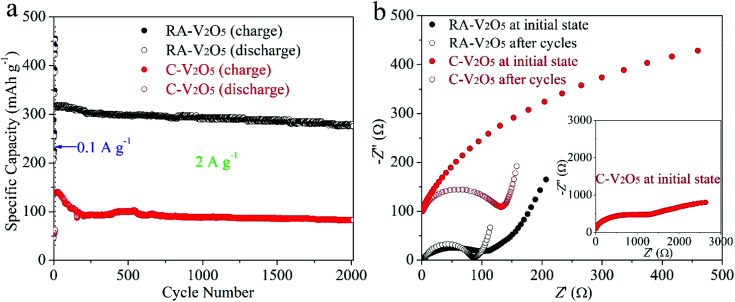
(a) Cycling performances of RA-V_2_O_5_ and C-V_2_O_5_ at 2 A g^−1^, conducted after 10 cycles at 0.1 A g^−1^. (b) Nyquist plots of RA-V_2_O_5_ and C-V_2_O_5_ at initial state and after 1000 cycles at 2 A g^−1^.

Cycling stability is critical for electrode materials. Fig. 5a shows charge and discharge capacities of RA-V_2_O_5_ and C-V_2_O_5_ during 2000 cycles at 2 A g^−1^. The cycling performance tests were conducted after 10 cycles at 0.1 A g^−1^. It is seen that the charge capacity is nearly identical to the discharge one at each cycle, implying high coulombic efficiency. In the respect of discharge, RA-V_2_O_5_ offers an initial capacity of 317.6 mA h g^−1^, which gradually drops to 293.2 and 275.6 mA h g^−1^ at the 1000^th^ and 2000^th^ cycle, respectively. That is, the capacity retention of RA-V_2_O_5_ is up to 92.3% and 86.8% after 1000 and 2000 cycles, respectively. As for C-V_2_O_5_, its discharge capacity is merely 82.8 mA h g^−1^ at the 2000^th^ cycle, and the corresponding capacity retention is merely 59.4%. After around 30 days (including the period of cycling at 2 A g^−1^ for 2000 cycles), the electrolyte of RA-V_2_O_5_ coin cells was collected by washing the electrodes, separators, and coin cell shells with deionized water. Then the collected electrolyte was subjected to ICP-MS analysis, which reveals only trace amount of vanadium (0.0288%) from RA-V_2_O_5_ can be dissolved. Therefore, the active material dissolution is not the main reason of capacity fading for V_2_O_5_ cathode materials. In order to provide more insight into the electrochemical kinetics of RA-V_2_O_5_, EIS measurements were performed using 5 mV amplitude with frequency ranging from 10 mHz to 100 kHz. As is shown in Fig. 5b, the Nyquist plots of RA-V_2_O_5_ consist of two parts, *i.e.* a depressed semicircle in the high frequency region (charge transfer process) and a sloped line in the low frequency region (diffusion-limited process).^52^ At the initial state, the impedances of RA-V_2_O_5_ are considerably lower than that of C-V_2_O_5_, illustrating the morphology engineering adopted by the present study can significantly enhance electrochemical kinetics. Specifically, the charge transfer resistance (*R*_ct_) of RA-V_2_O_5_ is merely 103.1 Ω, which gets even smaller (66.8 Ω) at the end of 1000 cycles. The reduction of *R*_ct_ might result from activation mentioned in the above section.

**Fig. 6:**
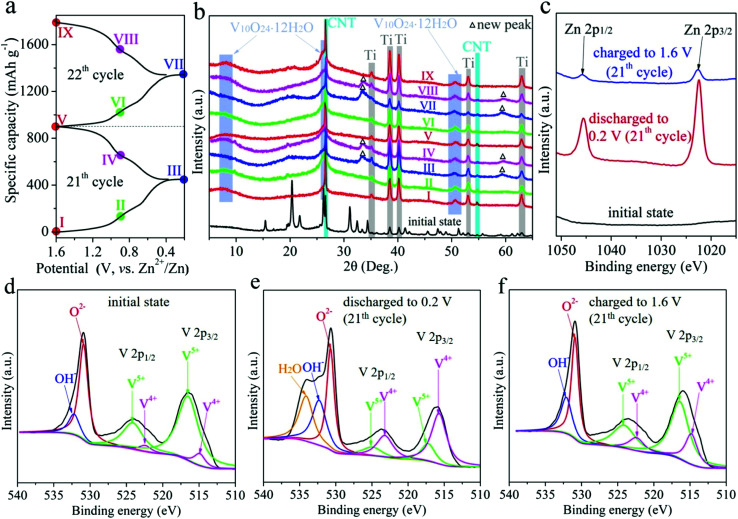
(a) The GCD curve of RA-V_2_O_5_ at 21^th^ and 22^th^ cycles at 0.1 A g^−1^, presenting nine different charge/discharge states. (b) The *ex situ* XRD patterns of RA-V_2_O_5_ at different states. The *ex situ* high-resolution (c) Zn 2p and (d–f) V 2p XPS spectra of RA-V_2_O_5_ at the initial state, 0.2 V (21^th^ cycle), and 1.6 V (21^th^ cycle).

The charge storage mechanism of RA-V_2_O_5_ was investigate using *ex situ* XRD measurements. The RA-V_2_O_5_ electrodes were cycled for 20 times at 0.1 A g^−1^ to reach a stable state. Subsequently during the 21^th^ and 22^th^ cycles, the RA-V_2_O_5_ electrodes were discharged or charged to a certain state (marked in Fig. 6a). After that, the RA-V_2_O_5_ electrodes were separated from the coin cells, rinsed with distilled water thoroughly, and dried under vacuum at room temperature. Herein, carbon black conductive agent was replaced by carbon nanotubes (CNT), so as to avoid the cracking of electrodes during washing with water. Fig. 6b shows XRD patterns of RA-V_2_O_5_ at different states. Except for the peaks originating from the Ti current collector and CNT conductive agent (the XRD pattern of neat CNT is shown in Fig. S8†), all the peaks of the electrode at the initial state (before cycles) can be indexed as the shcherbinaite V_2_O_5_. Surprisingly, all the peaks belonging to V_2_O_5_ disappear after 20 cycles. Instead, several new humps appear and match with V_10_O_24_·12H_2_O.^53^ These new peaks are rather broad and very low in intensity, indicating high-crystalline V_2_O_5_ is electrochemically transformed into amorphous V_10_O_24_·12H_2_O. When the electrode is discharged to 0.2 V, two new peaks are observed, and then disappear at 1.6 V. These new peaks coincide with the ones for Zn_0.25_V_2_O_5_·*n*H_2_O at discharged states.^23^ In Fig. S9,† some peaks/humps are magnified. It is seen the hump centered at *ca.* 50.4° (corresponding to V_10_O_24_·12H_2_O) moves to lower degree during discharging and shifts to higher degree during charging, due to the (de)intercalation of Zn^2+^ ions and H_2_O molecules. The above results indicate high-crystalline V_2_O_5_ is electrochemically transformed into amorphous V_10_O_24_·12H_2_O after prolonged cycles. This phenomenon has not been reported for vanadium-based oxide materials before, probably due to that previous reports mainly focused on initial several cycles. In fact, activation process often exists for vanadium-based oxide materials, especially for V_2_O_5_.^32,34,37,40^ In the present study, the rod-like morphology of RA-V_2_O_5_ is found to collapse after 2000 cycles at 2 A g^−1^, and in the meantime the nanosheets evolve into interconnected nanowires, as shown in Fig. S10.† Herein, the phase transformation and morphology evolution might account for the activation process of V_2_O_5_.

What's more, the *ex situ* XPS measurements were conducted on RA-V_2_O_5_ at the 21^th^ cycle, as shown in Fig. 6c–f, in which the spectra were calibrated using the C 1s peak at 284.8 eV. In Fig. 6c, Zn is absent at the initial state. When the electrode is discharged to 0.2 V, two strong peaks corresponding to Zn 2p appear, confirming Zn^2+^ is inserted into the host material. When the RA-V_2_O_5_ electrode is charged back to 1.6 V, these two peaks are still present, whereas their intensity is far below that for 0.2 V, indicative of good reversibility. The above result also demonstrates that a small amount of inserted Zn^2+^ ions cannot be extracted, agreeing well with previous reports.^24,42^ This might be the origin of capacity decay. As can be seen from Fig. 6d–f, the V^5+^ peak becomes weaker and the V^4+^ peak gets stronger when the electrode is discharged, indicating that part of V^5+^ is reduced to V^4+^ for charge balance. This process is reversible since V^4+^ can be oxidized back to V^5+^ at 1.6 V. It is also seen that the O 1s subpeak that is assigned to H_2_O is enhanced significantly when the electrode is discharged to 0.2 V, suggesting that H_2_O molecules are incorporated into the host material together with Zn^2+^ ion intercalation.^26,28,53^ During the charge process, these H_2_O molecules are released. The incorporated H_2_O molecules within the lattice of host material can effectively shield the electrostatic field of Zn^2+^ ions that are inserted at the same time, therefore beneficial to electrochemical kinetics.

## 4. Conclusions

Success development of AZIBs mainly depends on the design of cathode materials. In this work, we highlight the importance of morphology engineering by fabricating RA-V_2_O_5_*via* decomposing MIL-47 at 350 °C in air. Interestingly, RA-V_2_O_5_ rods are assembled by tiny nanosheets. Such intriguing morphology and structure endow RA-V_2_O_5_ with great performances as the cathode material for AZIBs. It is noticed that the pseudocapacitive Zn^2+^ ion intercalation into RA-V_2_O_5_ occupies nearly or more than half of the total capacity, *e.g.*, 71.8% at 1 mV s^−1^. RA-V_2_O_5_ also possesses high Zn^2+^ ion diffusion coefficient of 10^−9^ to 10^−11^ cm^2^ s^−1^ and low charge transfer resistance around 100 Ω. The fundamental Zn^2+^ ion storage mechanism is also elaborated. The crystalline V_2_O_5_ is converted to amorphous V_10_O_24_·12H_2_O and the morphology changes a lot after cycles, which might account for the activation process of V_2_O_5_. And it is found the Zn^2+^ ion (de)intercalation is accompanied by the reversible uptake/release of H_2_O molecules and redox transitions between V^5+^ and V^4+^. In summary, this work offers a new avenue for designing high-performance cathode materials of AZIBs and reveals a new phenomenon associated with Zn^2+^ ion storage.

## Conflicts of interest

There are no conflicts to declare.

## Supplementary Material

RA-009-C9RA06143F-s001
