# Supplementary material for: Rod-like anhydrous V2O5 assembled by tiny nanosheets as a high-performance cathode material for aqueous zinc-ion batteries
Source: RSC Adv. 2019 Sep 26;9(52):30556–64. doi: 10.1039/c9ra06143f (PMC9072160; doi:10.1039/c9ra06143f)
Supplement: RA-009-C9RA06143F-s001 [file RA-009-C9RA06143F-s001.pdf]

Electronic Supplementary Material (ESI) for RSC Advances.
This journal is © The Royal Society of Chemistry 2019

Electronic Supplementary Information (ESI)

Rod-like anhydrous V_2O_5 assembled by tiny nanosheets as a high-performance cathode material for aqueous zinc-ion batteries

Weijun Zhou,[a] Jizhang Chen,*[a] Minfeng Chen,[a] Xinwu Xu,[a] Qinghua Tian,[b] Junling Xu*[c] and Ching-Ping Wong[c,d]

[a] *College of Materials Science and Engineering, Nanjing Forestry University, Nanjing 210037, China*

[b] *Department of Chemistry, School of Sciences, Zhejiang Sci-Tech University, Hangzhou 310018, China*

[c] *Department of Electronic Engineering, The Chinese University of Hong Kong, NT, Hong Kong, China*

[d] *School of Materials Science and Engineering, Georgia Institute of Technology, Atlanta, United States*

E-mail addresses: jizhang.chen@hotmail.com (J. Chen) and junlingxu@outlook.com (J. Xu)

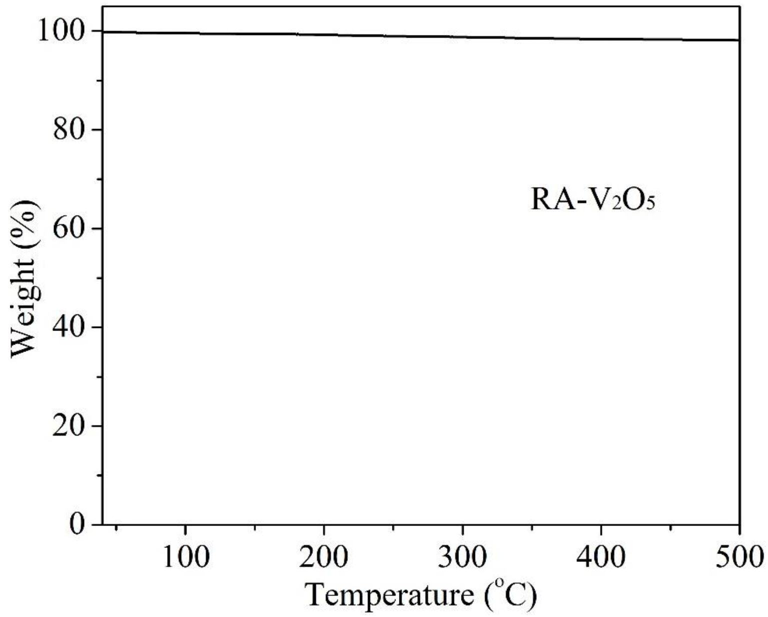

Fig. S1 TGA curve of RA-V_2O_5, measured in air at a heating rate of 10 °C min^{-1}.

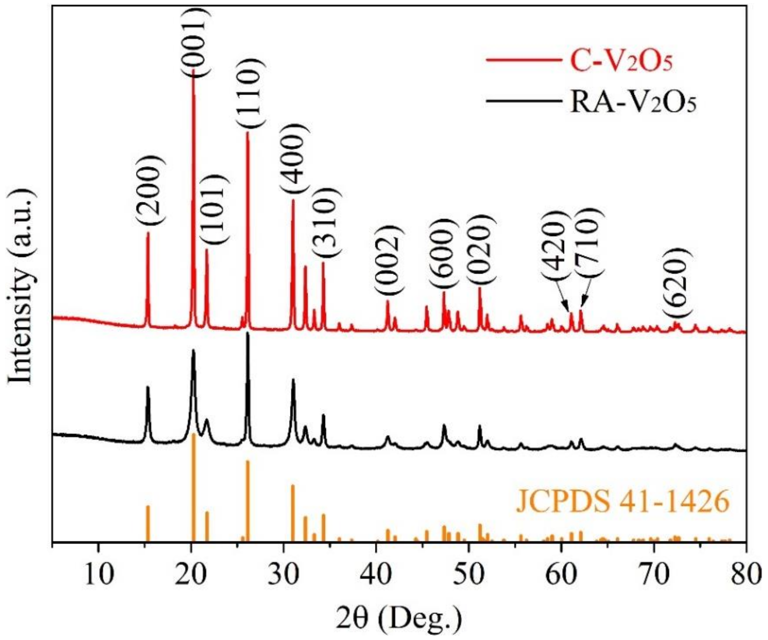

Fig. S2 XRD pattern of C-V_2O_5, in comparison with that of RA-V_2O_5.

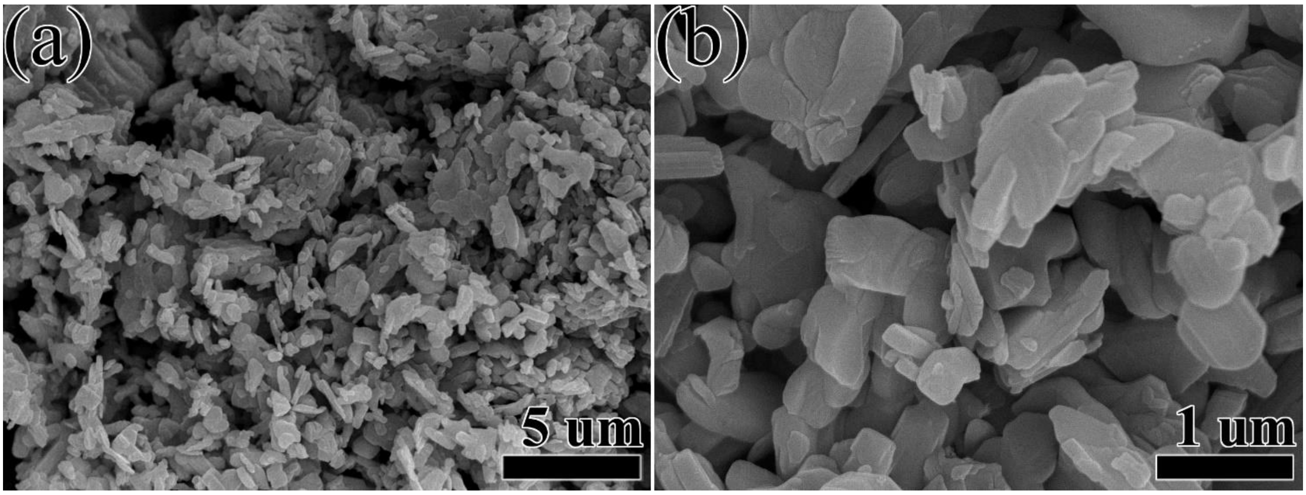

Fig. S3 (a, b) SEM images C-V_2O_5.

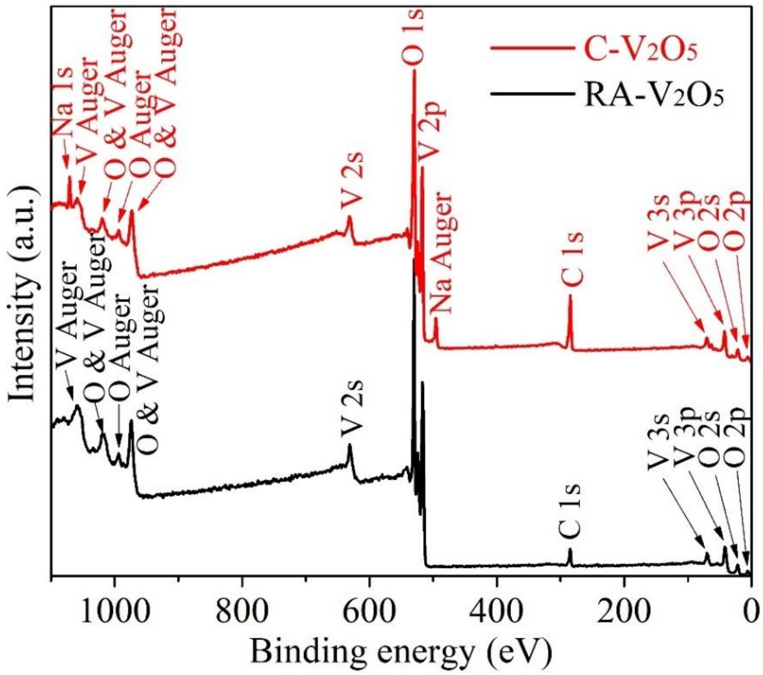

Fig. S4 XPS spectra of RA-V_2O_5 and C-V_2O_5.

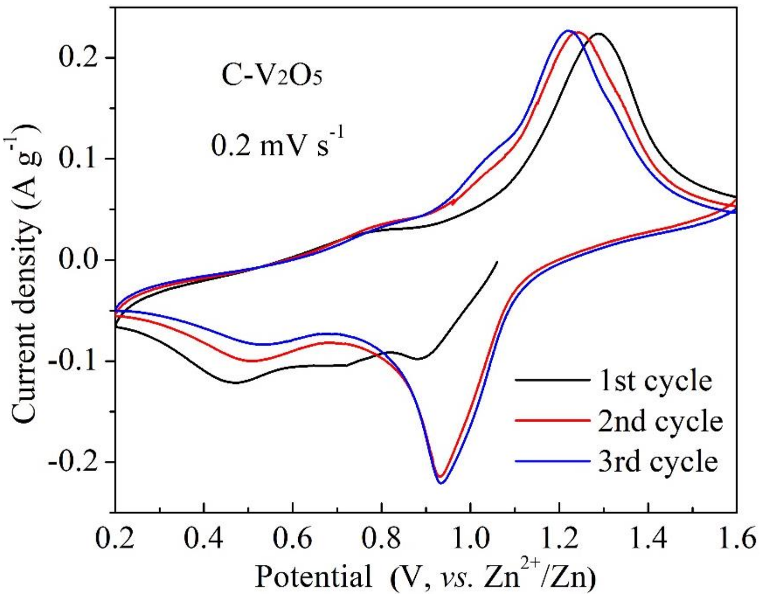

Fig. S5 CV curves of C-V_2O_5 in initial three cycles at 0.2 mV s^{-1}.

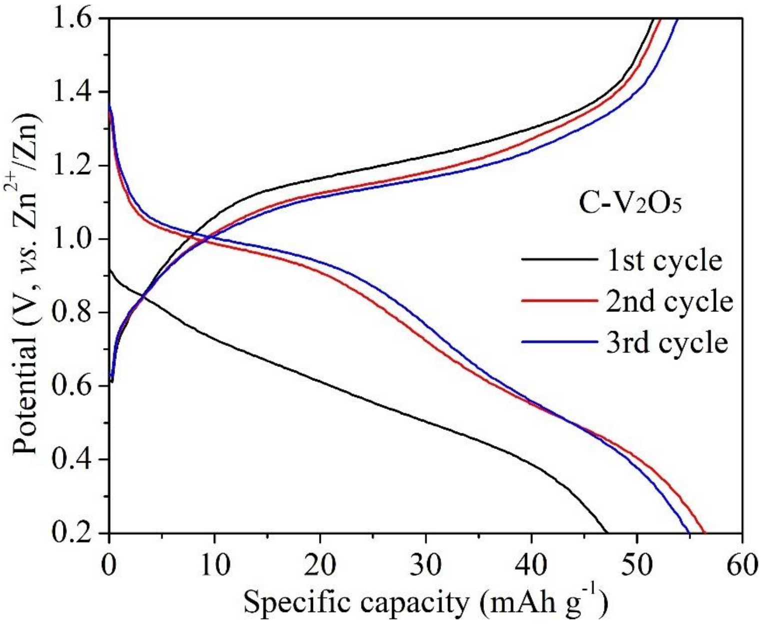

Fig. S6 GCD curves of C-V_2O_5 in initial three cycles at 0.1 A g^{-1}**.**

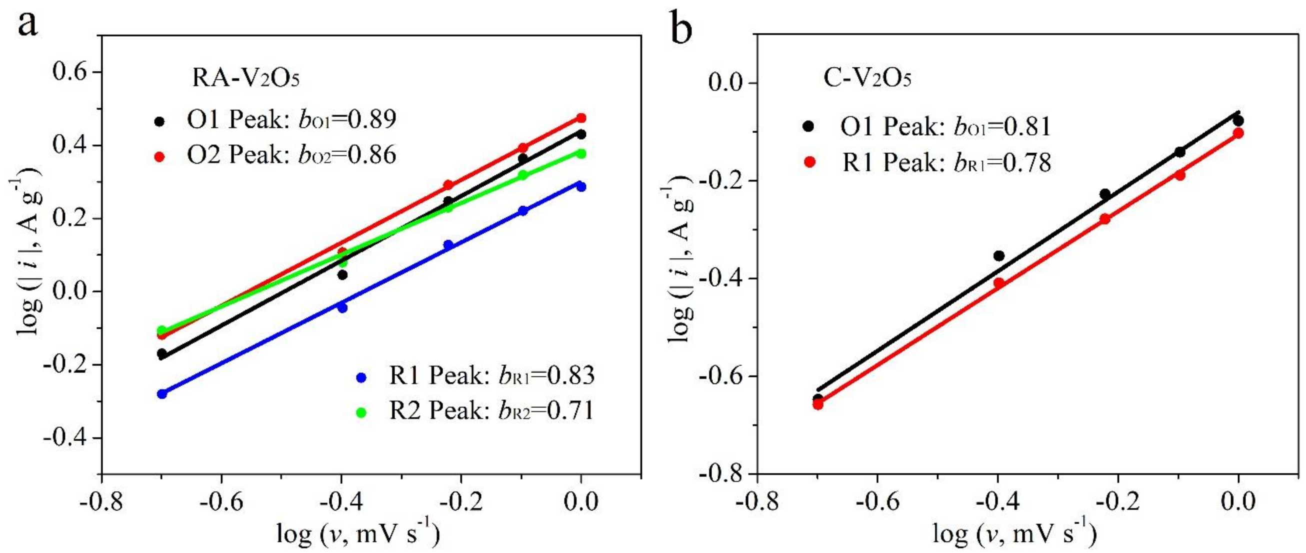

Fig. S7 Log (*i*) versus log (*v*) plots of different redox peaks of (a) RA-V_2O_5 and (b) C-V_2O_5 under CV measurements.

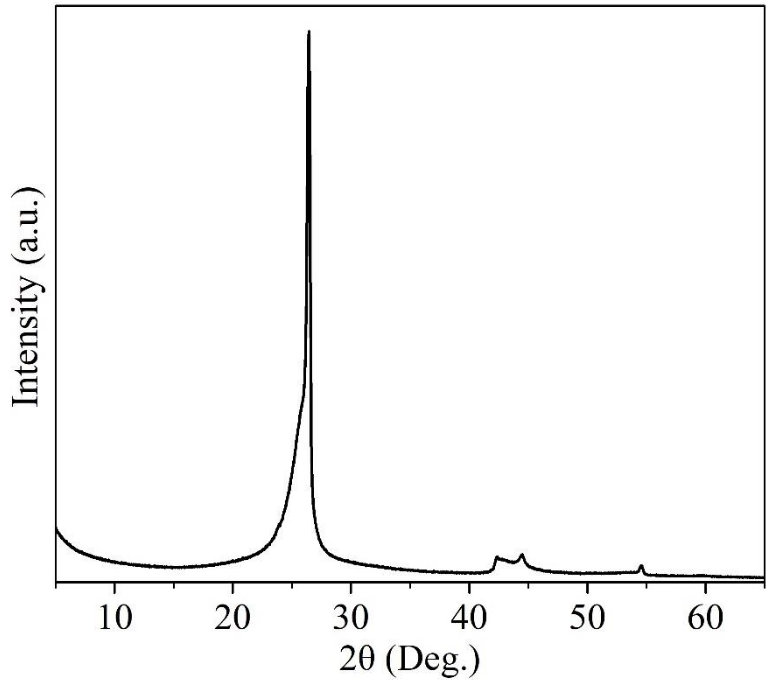

Fig. S8 XRD pattern of neat CNT power.

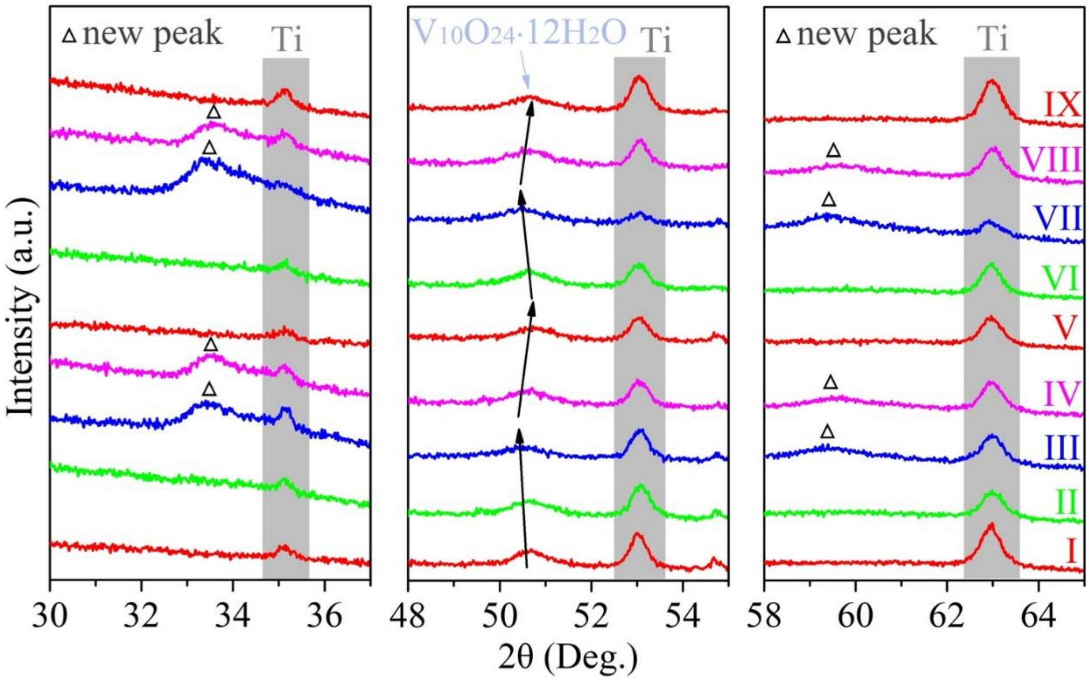

Fig. S9 The magnified ex situ XRD patterns of RA-V_2O_5 at nine different charge/discharge states at 21th and 22th cycles.

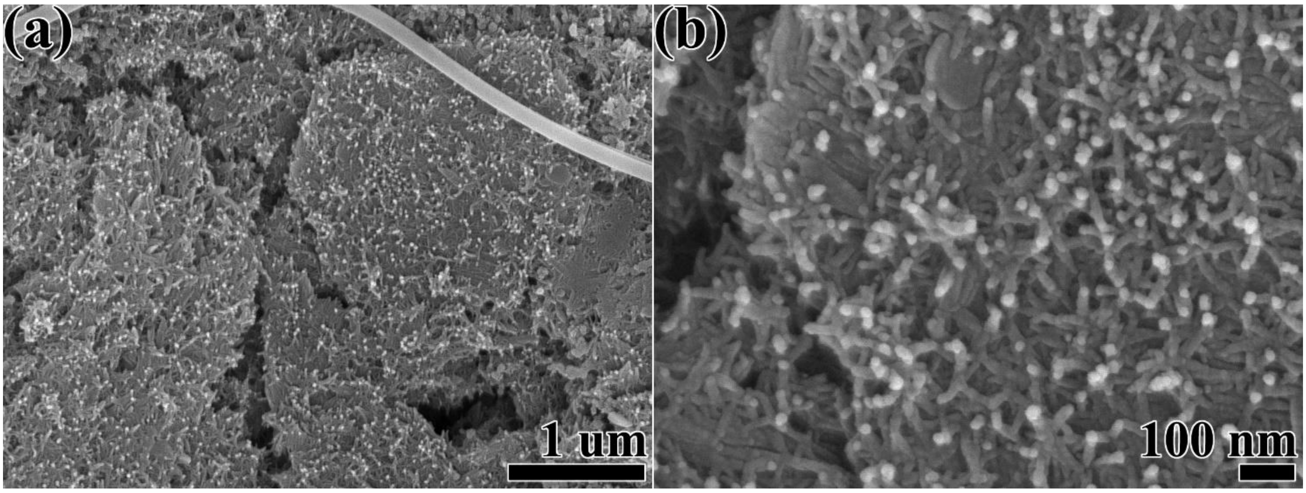

Fig. S10 SEM images of the RA-V_2O_5 electrode surface after 2000 cycles at 2 A g^{-1}.

Table S1 The capacities of RA-V_2O_5 in comparison with that of state-of-the-art vanadium-based cathode materials for AZIBs.

Cathode material	Capacity	Reference
RA-V_2O_5	449.8 mA h g^{-1} at 0.1 A g^{-1}<br>314.3 mA h g^{-1} at 2 A g^{-1}<br>186.8 mA h g^{-1} at 5 A g^{-1}	This report
$Mg_{0.34}V_2O_5·0.84H_2O$	353 mA h g^{-1} at 0.1 A g^{-1}<br>81 mA h g^{-1} at 5 A g^{-1}	1
$Ag_{0.4}V_2O_5$	340 mA h g^{-1} at 0.1 A g^{-1}<br>185 mA h g^{-1} at 2 A g^{-1}	2
Porous V_2O_5 nanofibers	319 mA h g^{-1} at 0.02A g^{-1}<br>104 mA h g^{-1} at 3 A g^{-1}	3
V_2O_5 nanosheets	224 mA h g^{-1} at 0.1 A g^{-1}<br>100 mA h g^{-1} at 2 A g^{-1}	4
V_2O_5 nanospheres	188.7 mA h g^{-1} at 0.5 A g^{-1}<br>138.3 mA h g^{-1} at 5 A g^{-1}	5
V_2O_5 hollow spheres	280 mA h g^{-1} at 0.2 A g^{-1}<br>147 mA h g^{-1} at 5 A g^{-1}	6
VO_2	283 mA h g^{-1} at 0.1 A g^{-1}<br>72 mA h g^{-1} at 5 A g^{-1}	7
VO_2	274 mA h g^{-1} at 0.1 A g^{-1}<br>170 mA h g^{-1} at 5 A g^{-1}	8
$V_{10}O_{24}·12H_2O$	164.5 mA h g^{-1} at 0.2 A g^{-1}<br>90.4 mA h g^{-1} at 5 A g^{-1}	9
VS_2	190.3 mA h g^{-1} at 0.05 A g^{-1}<br>115.5 mA h g^{-1} at 2 A g^{-1}	10
LiV_3O_8	230 mA h g^{-1} at 0.033 A g^{-1}<br>29 mA h g^{-1} at 1.666 A g^{-1}	11
$NaV_3O_8·1.5H_2O$	375 mA h g^{-1} at 0.1 A g^{-1}<br>165 mA h g^{-1} at 4 A g^{-1}	12
NaV_6O_{15} nanorods	427 mA h g^{-1} at 0.05 A g^{-1}<br>195 mA h g^{-1} at 1.6 A g^{-1}	13
$Zn_2V_2O_7$	203.4 mA h g^{-1} at 0.3 A g^{-1}<br>155 mA h g^{-1} at 4 A g^{-1}	14
$Zn_2(OH)VO_4$	204 mA h g^{-1} at 0.1 A g^{-1}<br>160 mA h g^{-1} at 2 A g^{-1}	15
$Zn_3V_2O_7(OH)_2·2H_2O$	213 mA h g^{-1} at 0.05 A g^{-1}<br>76 mA h g^{-1} at 3 A g^{-1}	16
$Fe_5V_{15}O_{39}(OH)_9·9H_2O$	385 mA h g^{-1} at 0.1 A g^{-1}<br>105 mA h g^{-1} at 5 A g^{-1}	17
$VOPO_4$	139 mA h g^{-1} at 0.05 A g^{-1}<br>50 mA h g^{-1} at 5 A g^{-1}	18

References

1. F. W. Ming, H. F. Liang, Y. J. Lei, S. Kandambeth, M. Eddaoudi and H. N. Alshareef, *ACS Energy Lett.*, 2018, **3**, 2602-2609.
2. L. Shan, Y. Yang, W. Zhang, H. Chen, G. Fang, J. Zhou and S. Liang, *Energy Storage Mater.*, 2018, **18**, 10-14.
3. X. Chen, L. Wang, H. Li, F. Cheng and J. Chen, *J. Energy Chem.*, 2019, **38**, 20-25.
4. J. Zhou, L. T. Shan, Z. X. Wu, X. Guo, G. Z. Fang and S. Q. Liang, *Chem. Commun.*, 2018, **54**, 4457-4460.
5. F. Liu, Z. Chen, G. Fang, Z. Wang, Y. Cai, B. Tang, J. Zhou and S. Liang, *Nano-Micro Lett.*, 2019, **11**, 25.
6. H. Qin, L. Chen, L. Wang, X. Chen and Z. Yang, *Electrochim. Acta*, 2019, **306**, 307-316.
7. L. N. Chen, Y. S. Ruan, G. B. Zhang, Q. L. Wei, Y. L. Jiang, T. F. Xiong, P. He, W. Yang, M. Y. Yan, Q. Y. An and L. Q. Mai, *Chem. Mater.*, 2019, **31**, 699-706.
8. T. Y. Wei, Q. Li, G. Z. Yang and C. X. Wang, *J. Mater. Chem. A*, 2018, **6**, 8006-8012.
9. T. Y. Wei, Q. Li, G. Z. Yang and C. X. Wang, *Electrochim. Acta*, 2018, **287**, 60-67.
10. P. He, M. Y. Yan, G. B. Zhang, R. M. Sun, L. N. Chen, Q. Y. An and L. Q. Mai, *Adv. Energy Mater.*, 2017, **7**, 1601920.
11. M. H. Alfaruqi, V. Mathew, J. Song, S. Kim, S. Islam, D. T. Pham, J. Jo, S. Kim, J. P. Baboo, Z. Xiu, K. S. Lee, Y. K. Sun and J. Kim, *Chem. Mater.*, 2017, **29**, 1684-1694.
12. F. Wan, L. L. Zhang, X. Dai, X. Y. Wang, Z. Q. Niu and J. Chen, *Nat. Commun.*, 2018, **9**, 1656.
13. S. Islam, M. H. Alfaruqi, B. Sambandam, D. Y. Putro, S. Kim, J. Jo, S. Kim, V. Mathew and J. Kim, *Chem. Commun.*, 2019, **55**, 3793-3796.
14. B. Sambandam, V. Soundharrajan, S. Kim, M. H. Alfaruqi, J. Jo, S. Kim, V. Mathew, Y. K. Sun and J. Kim, *J. Mater. Chem. A*, 2018, **6**, 3850-3856.
15. D. L. Chao, C. Zhu, M. Song, P. Liang, X. Zhang, N. H. Tiep, H. F. Zhao, J. Wang, R. M. Wang, H. Zhang and H. J. Fan, *Adv. Mater.*, 2018, **30**, 1803181.
16. C. Xia, J. Guo, Y. J. Lei, H. F. Liang, C. Zhao and H. N. Alshareef, *Adv. Mater.*, 2018, **30**, 1705580.
17. Z. Peng, Q. L. Wei, S. S. Tan, P. He, W. Luo, Q. Y. An and L. Q. Mai, *Chem. Commun.*, 2018, **54**, 4041-4044.
18. F. Wan, Y. Zhang, L. Zhang, D. Liu, C. Wang, L. Song, Z. Niu and J. Chen, *Angew. Chem. Int. Edit.*, 2019, **58**, 7062-7067.
